# Molecular deconvolution of the neutralizing antibodies induced by an inactivated SARS-CoV-2 virus vaccine

**DOI:** 10.1007/s13238-021-00840-z

**Published:** 2021-04-28

**Authors:** Xingdong Zhou, Hui Wang, Qun Ji, Mingjuan Du, Yuexia Liang, Huanhuan Li, Fan Li, Hang Shang, Xiujuan Zhu, Wei Wang, Lichun Jiang, Alexey V. Stepanov, Tianyu Ma, Nanxin Gong, Xiaodong Jia, Alexander G. Gabibov, Zhiyong Lou, Yinying Lu, Yu Guo, Hongkai Zhang, Xiaoming Yang

**Affiliations:** 1 State Key Laboratory of Medicinal Chemical Biology and College of Life Sciences, Nankai University, Tianjin, 300071 China; 2 Beijing Institute of Biological Products Company Limited, Beijing, 100176 China; 3 Shanghai Institute for Advanced Immunochemical Studies, ShanghaiTech University, Shanghai, 201210 China; 4 M.M. Shemyakin and Yu.A. Ovchinnikov Institute of Bioorganic Chemistry of the Russian Academy of Sciences, Moscow, Russia 117997; 5 Comprehensive Liver Cancer Center, The 5th Medical Center of PLA General Hospital, Beijing, 100039 China; 6 MOE Key Laboratory of Protein Science & Collaborative Innovation Center of Biotherapy, School of Medicine, Tsinghua University, Beijing, 100084 China

**Dear Editor,**

The rapid emergence and persistence of the pandemic caused by severe acute respiratory syndrome coronavirus 2 (SARS-CoV-2) has had enormous impacts on global health and the economy. Effective vaccines against SARS-CoV-2 are urgently needed to control the coronavirus disease 2019 (COVID-19) pandemic, and multiple vaccines have been found to be efficacious in preventing symptomatic COVID-19(Polack et al., 2020; Wu et al., 2020; Jones and Roy, 2021). We have developed a traditional beta-propiolactone-inactivated aluminum hydroxide-adjuvanted whole-virion SARS-CoV-2 vaccine (BBIBP-CorV), which elicited protective immune responses in clinical trials (Wang et al., 2020; Xia et al., 2021). The vaccine has been granted conditional approvals or emergency use authorizations (EUAs) in China and other countries.

The spike protein (S protein) is the main target of the humoral response during SARS-CoV-2 vaccination. The S protein is located on the surface of the SARS-CoV-2 virion, is involved in the entry step of virus infection and consists of two subdomains: the N-terminal S1 domain, which contains the N-terminal domains (NTDs) and the receptor-binding domain (RBD) that recognizes the host cell receptor angiotensin-converting enzyme 2 (ACE2), and the S2 domain responsible for fusion between the virus and cell membranes. Philip J. M. Brouwer isolated monoclonal antibodies from three convalescent COVID-19 patients using a SARS-CoV-2 spike protein and revealed that the SARS-CoV-2 spike protein contains multiple distinct antigenic sites, which could provide guidance for vaccine design (Brouwer et al., 2020).

The serological response after viral infection or vaccination is composed of a mixture of antibodies against different antigenic domains of the virus. Currently, serological assays are used to monitor the antibody response following vaccination (Anderson et al., 2020; Wang et al., 2020). Molecular deconvolution of the antibody repertoire after vaccination could provide a more complete understanding of the effectiveness and mechanism of the vaccines than conventional methods.

Cloning of individual B cells isolated by fluorescence-activated cell sorting (FACS) has been used extensively to discover neutralizing antibodies from convalescent patients who have recovered from infections (Wen et al., 2020). Potent neutralizing antibodies that bind to the S protein of SARS-CoV-2 have been identified using these methods (Ju et al., 2020). SARS-CoV-2-neutralizing antibodies were also discovered by single-cell VDJ sequencing of antigen-enriched B cells from convalescent patients (Cao et al., 2020). The single-cell sequencing method allows simultaneous acquisition of B cell receptor (BCR) sequences and transcriptomic information, with the cognate heavy and light chains of antibodies determined bioinformatically. The selected antibodies need to be synthesized and expressed for further characterization, which is well suited for fast antibody identification and development.

Recently, a microfluidics-based technology was developed to physically link the variable region of the heavy chain (VH) and variable region of the light chain (VL) from the same B cell (Wang et al., 2018). The resulting natively paired VH:VL antibody library can be directly screened using phage display or yeast display to isolate antibody clones specific to different antigens (Lerner, 2006). This method has been used to discover antiinfection antibodies, including broadly neutralizing antibodies (bNAbs) specific to HIV-1, Ebola virus and influenza virus (Rajan et al., 2018; Wang et al., 2018). In addition, as complete sets of VH and VL genes are preserved in their natural pairing, this method is well suited for characterization of the immune repertoire. Two individuals (Table. S1) with no prior SARS-CoV-2 infection history were vaccinated with the two-dose SARS-CoV-2 vaccine BBIBP-CorV, and blood was collected two months after the 2nd dose of vaccine (Fig. 1A). Plasma from both donors demonstrated strong binding to the SARS-CoV-2 S protein and effective neutralizing activity against 2 strains of live SARS-CoV-2 (Fig. 1B and 1C).

**Figure 1:**
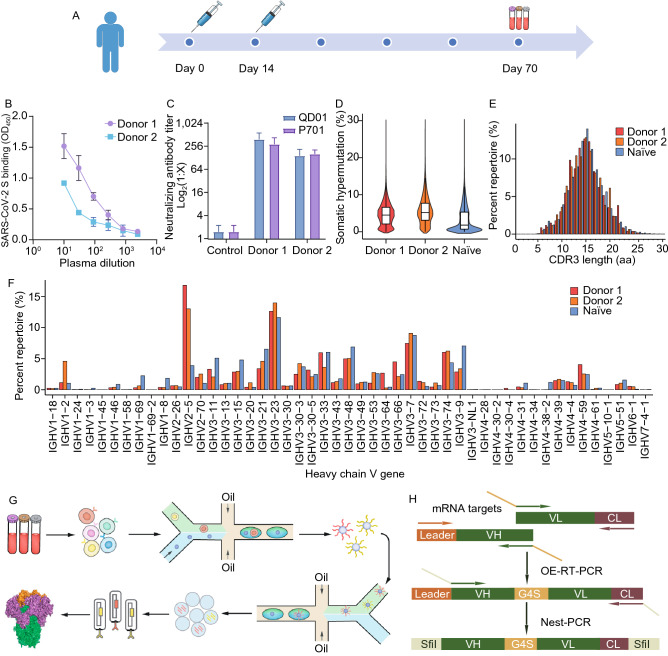
**SARS-CoV-2-specific response in human vaccination**. (A) Immunization and blood collection regimen. (B) Binding of plasma from donor 1 and donor 2 to SARS-CoV-2 S protein, as determined by ELISA. The mean values and SDs of three technical replicates are shown. (C) Neutralization of two SARS-CoV-2 strains (QD01 and P701) by plasma from donor 1 and donor 2. The mean values and SDs of two technical replicates are shown. (D) Violin plot showing SHM levels (nucleotides) of each donor. The lower, middle and upper edges of the boxplots represent the 25th, 50th and 75th percentiles, respectively. (E) Distribution of heavy chain CDR3 lengths in B cells from vaccinated and naïve donors. (F) Bar graph showing VH germline usage (%) in vaccinated and naïve donors. (G) Outline of microfluidics-based construction of a natively paired VH:VL antibody repertoire. Isolated B cells were purified from blood samples and encapsulated into water-in-oil droplets with beads for mRNA capture. mRNA-captured beads and RT-PCR reagents were reencapsulated, resulting in an amplicon-derived scFv library that can be screened by phage display technology. (H) Schematic of OE-PCR to construct natively paired VH:VL antibody libraries. VH and VL from each encapsulated B cell mRNA are amplified with specific primer sets and paired in-frame via complementary overhangs (yellow). A nested PCR with VH and CL primers generates full-length scFv with SfiI restriction sites for subcloning into phagemid vectors for library generation

We investigated the B cell response to vaccination by sequencing heavy chain variable regions of antibodies. The mean somatic hypermutation (SHM, or germline divergence) of donor 1 and donor 2 was 4.68% and 5.69%, respectively (Fig. 1D). The SHM of naïve donors was 3.25%, which was lower than those of both donors (two-sample Kolmogorov-Smirnov tests, *P* < 0.001 for both). The mean heavy chain complementarity-determining region 3 (CDRH3) lengths of donor 1 and donor 2 (14.11 and 14.01 amino acids, respectively) were similar to that of a naïve donor (14.00 amino acids) (Fig. 1E). Both vaccinated donors and the naive donor exhibited a similar distribution of germline V gene usage, except that IGVH2-5 was enriched in the vaccinated donors (Fig. 1F).

We constructed a natively paired VH:VL antibody library from both donors using a microfluidics droplet-based platform. The process was divided into 3 steps, as outlined in Fig. 1G and 1H. First, individual B cells were encapsulated into droplets with oligo-dT beads and lysis buffer using a microfluidics droplet system designed to merge two streams of aqueous fluids: one carrying a suspension of B cells and the other containing oligo-dT beads and lysis buffer. The density of B cells was adjusted such that one cell was distributed per ten to twenty droplets (Fig. S1A). For the second step, the mRNA-capture beads were purified from the droplets, and then individual beads were re-encapsulated into a second emulsion with reverse transcription (RT) and overlap-extension (OE)-PCR amplification mix (Fig. S1B). The VHs were amplified with forward-VH and reverse-CH primers, and VLs were amplified with forward-VL and reverse-CL primers (von Boehmer et al., 2016). To assemble the single-chain variable fragment (scFv), complementary overhangs were added to the reverse-CL and forward-VH primer sets to fuse the VH and VL domains with a flexible 4GS linker. Finally, the amplification products were recovered from the droplets, and nested PCR was performed to improve the specificity of the amplification and add restriction enzyme sites (Fig. 1H). The scFv amplicons were cloned into a phagemid for subsequent phage display screening. This approach allows physical linkage of VH and VL into a single amplicon to maintain the native pairing of VH and VL, as validated using two cells expressing different antibodies (Fig. S1C).

To delineate the antibody response against the S protein, the paired VH:VL scFv library was subjected to two rounds of phage display panning against the full-length S protein. Strong enrichment was obtained for the libraries from both donors (Table S2). Individual phage clones were picked, and their binding to RBD, NTD or S2 domain protein fragments was determined by phage enzyme-linked immunosorbent assay (ELISA). We identified 5, 4 and 7 antibodies recognizing the RBD, NTD and S2 domain, respectively. The antibodies were subcloned into mammalian cell expression vectors for expression in HEK293F cells. We characterized the binding of each antibody against the different S protein domains by ELISA. Most RBD-reactive antibodies demonstrated medium-to-strong binding, while NTD- and S2-reactive antibodies showed relatively weak binding (Fig. S2), suggesting that the RBD could be a good candidate for vaccine design.

Next, we evaluated the neutralization activity of the isolated antibodies using four strains of live SARS-CoV-2, of which 3 were isolated from patients and 1 was obtained from an environmental sample. Five RBD-binding antibodies (1-0106, 1-0108, 2-01H5, 2-0126 and 2-0139) and one NTD-binding antibody (1-11H2) showed neutralizing activities against all four SARS-CoV-2 strains (Fig. 2A, 2B and Table S3). Notably, 2-0126 and 2-0139 possessed identical heavy chain CDR sequences but differed in light chain CDRs by three amino acids, indicative of the same clonotype of the BCR.

**Figure 2:**
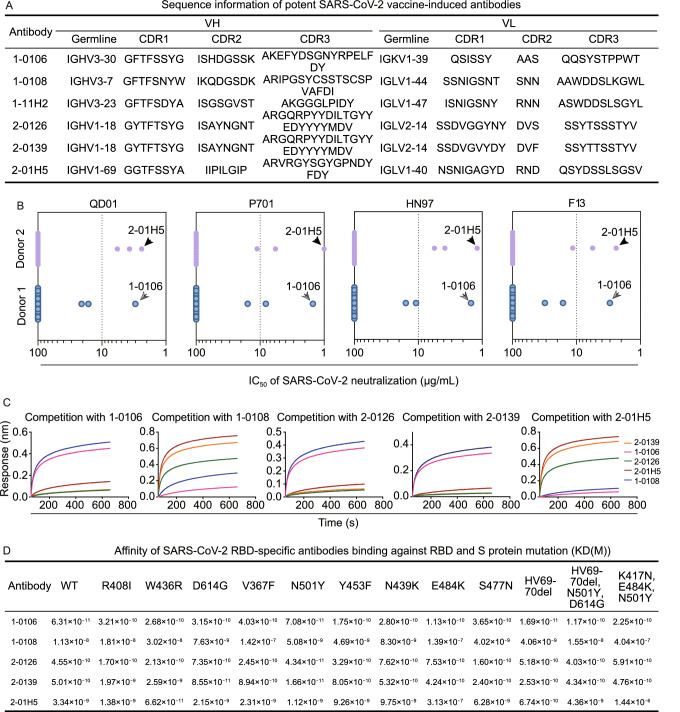
**Characterization of isolated S protein-reactive antibodies**. Sequence information of the identified neutralizing antibodies. 1-0106 is named for clone 1-0106 isolated from donor 1. Midpoint neutralization concentrations (IC_50_) for four SARS-CoV-2 strains (QD01/P701/HN97/F13). Each symbol represents an individual antibody. A competition assay was performed among different antibodies for binding to the RBD. The immobilized RBD was first incubated with 1-106, 1-108, 2-0126, 2-0139 or 2-01H5. The capture of the second antibodies was monitored by measuring further changes after injecting the second antibody in the presence of the first antibody. (D) Binding of each antibody to RBD and S protein mutants

To characterize the interrelationships between different antibodies, we performed cross-competition assays using an in-tandem bio-layer interferometry (BLI) assay. All RBD-binding antibodies could be divided into two competition clusters (1-0106, 2-0126, and 2-0139 belong to one cluster, and 1-0108 and 2-01H5 belong to the other cluster). Both clusters included antibodies from the two donors (Fig. 2C), suggesting common epitopes targeted by the humoral immune response in different individuals. The results corroborate previous observations that neutralizing antibodies can recognize distinct antigenic sites of the RBD (Ju et al., 2020); thus, these antibodies may have a synergistic effect in inhibiting virus infection. To further evaluate the ability of each antibody to compete with ACE2 for binding to the RBD, we performed a cell-based competition assay. The antigen-binding fragment (Fab) of antibodies 1-0106 from donor 1 and 2-0139 from donor 2 efficiently blocked the attachment of the SARS-CoV-2 RBD to ACE2-expressing HEK293FT cells, indicating that blocking ACE2 binding may be their primary mechanism of neutralization (Fig. S3).

The antibodies were quite different in terms of germline usage, indicating that different germlines can be used to generate neutralizing antibodies (Fig. 2A). We generated two-dimensional (2D) identity-divergence plots to visualize sequence identity to the reference antibody and sequence divergence from their putative germline V sequences, which is a common metric to identify antigen-driven antibody evolution (Wen et al., 2020). The antibody-like lineage was observed to form an island extending from the main population on the 2D plot, indicating that there are many somatic hypermutation variants of the identified antibodies (Fig. S4).

Recently, the emerging SARS-CoV-2 B.1.1.7 strain identified in the UK and B.1.351 strain found in South Africa have raised concerns regarding vaccine efficacy against these new strains because of their extensive mutations in the virus spike protein. In addition to D614G, B.1.1.7 contains 8 spike mutations, including two deletions (69-70del, 144del) in the NTD, one mutation (N501Y) in the RBD, and P681H. B.1.351 contains 9 spike mutations, including mutations (242-244del, R246I) in the NTD, three mutations (K417N, E484K, N501Y) in the RBD, and A701V. We tested the reactivity of the identified neutralizing antibodies against S protein variants containing individual mutations and 2 variants containing multiple mutations (Figs. 2D and S5-8). Overall, antibodies 1-0106, 2-0126 and 2-0139 retained their reactivity against all single amino acid mutants tested, while the binding of 1-0108 and 2-01H5 against some single amino acid mutants was weakened. One change of particular interest is the E484K mutation of the virus S protein, which reduced recognition by multiple reported RBD-targeting neutralizing antibodies (Wang et al., 2021). We also observed impaired binding of 1-0108 and 2-01H5 to the RBD E484K mutant. However, the antibodies 1-0106, 2-0126 and 2-0139 showed no significant reduction in their binding to this mutant. All 5 identified RBD-targeting antibodies retained their binding affinity against the spike protein containing multiple core mutations in B.1.1.7 (HV69-70del, N501Y, D614G). For the spike protein mutant containing multiple core mutations in B.1.351 from South Africa (K417N, E484K, N501Y), antibodies 1-0106, 2-0126 and 2-0139 preserved their binding abilities, while binding of antibodies 1-0108 and 2-01H5 against this mutant was reduced (>10 fold). Thus, our data suggests that after vaccination, the sera in these 2 individuals containing at least these 2 clusters of neutralizing antibodies could at least largely preserve their reactivity against identified spike protein mutants and could be potentially useful against new variants. Nevertheless, the effectiveness of this vaccine will continue to be tested against new variants as they emerge.

In summary, the detailed analysis of the B cell repertoire in response to the inactivated SARS-CoV-2 vaccine BBIBP-CorV provides a molecular-level understanding of the mechanism of the inactivated SARS-CoV-2 vaccine and may facilitate a paradigm shift for future vaccine development and evaluation. The methods herein can be applied to other vaccines and may be of broad interest to the vaccine research community. The findings of our study also support that inactivated vaccines can elicit diverse and broadly neutralizing antibodies.

## Supplementary Information

Below is the link to the electronic supplementary material.

Supplementary material 1 (PDF 5890 kb)
