# Supplementary material for: Molecular deconvolution of the neutralizing antibodies induced by an inactivated SARS-CoV-2 virus vaccine
Source: Protein Cell. 2021 Apr 28;12(10):818–23. doi: 10.1007/s13238-021-00840-z (PMC8080482; doi:10.1007/s13238-021-00840-z)
Supplement: 13238_2021_840_MOESM1_ESM — Supplementary File [file 13238_2021_840_moesm1_esm.pdf]

Supplementary File

Materials and methods

Viruses, cells and bacteria culture

All work with infectious SARS-CoV-2 was performed in the biosafety level 3 facility of Beijing Institute of Biological Products Company Limited, Beijing, China. Strains QD01, P701 and HN97 were isolated from bronchoalveolar lavage samples or patient throat swabs, and strain F13 was isolated from an environmental sample. Vero E6 cells, HEK293FT cells and ACE2-293FT cells were grown in Dulbecco's modified Eagle's medium (DMEM) (Gibco) supplemented with 10% fetal bovine serum (Gibco), penicillin (100 IU/mL) and streptomycin (100 IU/mL). HEK293F cells were suspended and cultured in FreeStyle™ 293 Expression Medium (Thermo Scientific). All cells were maintained in a CO_2 incubator at 37°C. The bacterial strain XL-1 Blue was grown in 2×YT medium and maintained in SOC medium after library transformation.

Human subjects

Peripheral blood samples were collected from vaccine donors with informed consent. Peripheral blood mononuclear cells (PBMCs) were isolated using human lymphocyte separation medium (TBD Sciences) according to the manufacturer's protocol. Plasma was collected for ELISA binding tests and neutralization tests. Total B cells were then isolated from PBMCs as described in the Human B Cell Enrichment Kit (Stem Cell). Two million cells were split into two parts: one million cells were processed for bioinformatics analysis by NGS, and the other million B cells were processed for microfluidics-based antibody isolation.

ELISA

Polystyrene microplates (Corning) were coated overnight with 2 μg/mL SARS-CoV-2 S protein (ACRO Biosystems). After washing with PBS containing 0.2% Tween 20 (Sigma Aldrich), the plates were blocked using 2% BSA (Solarbio Life Sciences) in PBST for 1 h at 37°C. After washing with PBST, serial dilutions of testing plasma were

added to each well and incubated at 37°C for 1 h. After washing with PBST, horseradish peroxidase (HRP)-conjugated anti-human IgG antibody (SouthernBiotech) was added at a dilution of 1:3000 and incubated at 37°C for 1 h. After washing, TMB single component substrate solution (Solarbio Life Sciences) was added to the microplate and incubated at room temperature for 20 min. Then, the absorbance was detected at 450 nm/630 nm. The data were analyzed using GraphPad Prism 7.0.

Neutralization assay

Plasma or antibody was diluted to the required concentration in a 2-fold series, and an equal volume of challenge virus solution containing 100 CCID50 virus was added. After neutralization in a 37°C incubator for 2 h, 1.0~2.5×10^5/mL Vero cells were added to the wells (0.1 mL/well) and cultured in a CO_2 incubator at 37°C for 4 days. The Spearman-Karber method (Ramakrishnan, 2016) of cytopathic effect (CPE) determination was used to calculate the neutralization endpoint, that is, the greatest dilution of serum or antibody that can protect 50% of cells from infection by challenge with 100 CCID50 virus.

NGS of the VH repertoire

RNA was extracted from one million B cells using RNAiso Plus (Takara). The variable region of heavy chain (VH) was amplified using a HiScript II One Step RT-PCR kit (Vazyme) with primer sets designed to anneal to the VH leader sequence and constant region and for maximal coverage of all human immunoglobulin sequences. Amplicons were gel-extracted and purified prior to library preparation. Samples were sequenced using a 2 × 250 bp Nova pe250 platform.

Analysis of NGS data for antibody repertoires

Paired-end reads were merged in CLC Genomics Workbench V11.0. Germline V and J genes were assigned using BLAST+ in SONAR V1.1 (Schramm et al., 2016), and unproductive sequences were discarded (missing assignments, out-of-frame junctions, or stop codons). Sequences with CDR3 lengths (aa) longer than 30 and VH germline

divergence above 0.3 were also discarded. In SONAR, V(D)J sequences were clustered at 99% nucleotide sequence identity, and only clusters of 2 or more sequences were retained. Sequences related to the lineages of the six antibodies 1-0106, 1-0108, 1-215B, 2-01H5, 2-0126 and 2-0139 were identified using identity-divergence plots in SONAR and manually checked. Typically, low coverage is not sufficient to call high-confidence variants. In the current study, we used a conservative cutoff to keep the clones with 2 or more read counts in order to track the rare neutralizing antibodies as much as possible.

Generation of natively paired VH:VL antibody repertoires.

First, one million B cells were resuspended in 250 μL encapsulation buffer (PBS containing and 16% OptiPrep Density Gradient medium (Stem Cell). Encapsulation was performed on a 2-reagent droplet generation fluorophilic chip (Dolomite, cat. no. 3200510). The microfluidic chip has two input channels for fluorocarbon oil (BioRad), one for the cell suspension and one for 25% oligo-dT beads (NEB) in cell lysis buffer (Adler et al., 2017). A pressure-focusing pump (Dolomite) was used to pump the liquids through the chip to generate droplets and encapsulate cells with beads. The droplet size was adjusted to a diameter of 45-50 μm. Emulsions were collected into microcentrifuge tubes on ice. The beads were extracted from the droplets using 1H,1H,2H,2H-perfluorooctanol (PFO, Sigma-Aldrich).

Second, mRNA-bound beads were re-encapsulated into droplets of approximately 30 μm with an overlap extension RT-PCR (OE-RT-PCR) mix. Encapsulation was performed on a junction PMSD chip (Hanguang, Suzhou). The OE-RT-PCR mix contained a HiScript II One-Step RT-PCR mixture (Vazyme), plus forward-VH primers designed to anneal to the leader of VH1-VH4, forward-VL primers designed to anneal to the leader of the VL gene, reverse-CH and reverse-CL primers. VH and VL chains were physically linked by overlapping 4GS-linker sequences included on the reverse-CH and forward-VL primers. The amplified DNA was recovered from the droplets and purified using a PCR Clean-up Kit (Macherey-Nagel). Because VH1-VH4-derived antibodies predominated in the repertoire, based on the VH repertoire NGS sequencing results, and the germlines of the most neutralizing antibodies isolated from

convalescent patients were assigned to VH1 to VH4, we designed primers specifically targeting VH1 to VH4 to increase the specificity of amplification. Therefore, this study analyzed only VH1-4-derived antibodies.

For nested PCR, the product from the last step was first electrophoresed on a 1.7% agarose gel, and then the region between 800 bp and 1200 bp was excised and purified using NucleoSpin Gel and a PCR Clean-up Kit (Macherey-Nagel). Nested PCR was performed using new primer sets designed to anneal to the FR1 region of VH and CL to increase the specificity and add restriction sites for cloning into phagemid. PCR products were run on a 1.2% agarose gel, and the band at 900 bp was excised and purified using NucleoSpin Gel and a PCR Clean-up Kit (Macherey-Nagel).

Phage display library construction and preparation

The DNA amplicons from nested PCR were digested and ligated into pCGMT3 phagemid and transformed by electroporation into *E. coli* XL1-Blue. After transformation, prewarmed SOC medium was added, and the culture was shaken for 1 h at 37°C, after which 2×YT medium containing 2% glucose, 50 μg/mL carbenicillin and 10 μg/mL tetracycline was added, and the culture was then shaken for an additional hour. Then, helper phage VCMS13 was added, and the culture was shaken for an additional 2 h. Kanamycin was added to 70 μg/mL, and the culture was incubated at 30°C overnight.

Phage display to isolate SARS-CoV-2 S protein binding antibodies.

The phage library was incubated with biotinylated SARS-CoV-2 S protein (Acro Biosystems) for 2 h at room temperature, and the phage-antigen complex was captured by Dynabeads M280 (Life Technologies). Bound phage was eluted using glycine-HCl (pH 2.2) for 10 min at room temperature, and the pH of the eluate was adjusted to pH 7.5 (neutralized) with Tris-HCl (pH 8.0). The eluted phage was used to infect XLI-Blue cells at OD600 = 0.5 for 30 min at 37°C, after which 50 mL of 2×YT medium containing 2% glucose, 50 μg/mL carbenicillin and 10 μg/mL tetracycline was added; the culture was then shaken for 1 h at 37°C. Further growth, phage preparation, and panning were

repeated as described above. After 2-3 rounds of panning, individual phage clones were amplified for phage ELISA. For ELISA, 100 ng NTD (Acro Biosystems), RBD or S2 domain (Acro Biosystems) protein was immobilized on 96-well plates overnight and blocked for 40 min with 4% M-PBST (PBS+4% skim milk). Following washing with PBST (PBS + 0.05% Tween-20), diluted phage supernatant was added, and phage bound to the coated antigens was detected using an anti-M13-HRP antibody (1:5000) and ABTS as substrate.

Antibody expression and purification

Phagemid DNA from positive clones was isolated and sequenced. Antibodies were cloned into mammalian expression vectors. The antibodies were expressed transiently in HEK293F cells. Cells were transfected at a density of 0.8-1.2 million cells/mL by addition of a mix of PEIMAX (1 μg/μL) with expression plasmids. Supernatants were harvested at four days post transfection. Antibodies were purified by affinity purification using a Protein A bead (Merck) suspension according to the manufacturer's protocol for gravity flow. These monoclonal antibodies were eluted with 50 mM glycine-HCl pH 2.7 and neutralized with 1 M Tris/HCl pH 8.5. Protein eluates were concentrated, and the buffer was exchanged to PBS using a protein ultrafiltration tube (Millipore) with a 30 kDa molecular weight cutoff. Protein concentrations were determined by BCA assay.

ACE2 competition assay

HEK293T cells were transfected with the extracellular domain of the ACE2-expressing plasmid. HEK293T cells displayed ACE2 at 4 h after transfection. Before assaying competition with ACE2, the biotinylated spike RBD protein was blocked by Fab or BSA (1:10) at 37°C for 1.5 h. Then, the cells were stained with the mixture at 4°C for 1 h. After washing three times with ice-cold FACS buffer, the cells were incubated with SA-PE (1:200 dilution, Thermo Fisher) and incubated at RT for 30 min. After the final wash, the cells were analyzed by flow cytometry (LSR Fortessa, BD).

Measurement of antibody affinity for S proteins

The binding kinetics of anti-RBD antibodies were determined by biolayer interferometry on a ForteBio Octet RED96 instrument (Sartorius AG). In brief, his-tag RBD or S1 protein (wild type or mutant) (Tab. S4) was immobilized onto a Ni-NTA biosensor (Forte Bio cat no 18-5019) at 1 µg/mL for 180 s, and biosensors were then equilibrated for 180 s in PBST (PBS+0.05% Tween). The association of serial dilutions of antibodies was measured over 420 s, followed by 600 s dissociation in PBST. All the data were analyzed by "Data Analysis 10.0" from ForteBio (Sartorius AG), and the curve fitting model was 1:1 for both association and dissociation. Reference samples were subtracted to correct for nonspecific baseline drift.

Epitope binning of antibodies

The Avi-RBD-immobilized SA biosensors were incubated with the first antibodies at 200 nM for 600 s to equilibrate and then submerged in 200 nM second antibodies for 600 s after 180 s baseline in PBST, followed by running with reference samples and buffer. The matrix data were exported by "Data Analysis 10.0" from ForteBio (Sartorius AG) for external analysis of the self-binding signal threshold. The nonoverlapping epitopes were indicated by a ≥100% signal threshold compared with the self-activity signal.

Table S1: Donor information.

Table S2: Enrichment of different rounds of phage display panning.

Table S3: IC_{50} of antibodies neutralizing different strains.

Table S4: Mutant list.

Figure S1: Optimization of the microfluidics-based construction of the natively paired VH:VL antibody repertoire

(A) Single-cell encapsulation using droplet microfluidics. GFP-expressing cells were encapsulated into droplets. (B) Single-bead encapsulation using droplet microfluidics. mRNA-capturing beads were encapsulated into droplets. (C) Validation of native chain pairing. Two cells expressing different antibodies were mixed, and the natively paired antibody library was constructed using microfluidics-based methods. The resulting amplicons were purified, and native pairing was assessed using primers specific to the heavy and light chain CDR3 of each antibody. Products were obtained only when paired primers from the same antibody were used.

Figure S2: Binding of the isolated antibodies to SARS-CoV-2 S protein, RBD, NTD and S2, as determined by ELISA.

Figure S3: Competition of the antibodies with ACE2 for S protein binding. The biotinylated spike RBD protein was incubated with the indicated Fab before adding to ACE2-expressing HEK293T cells. The cells were stained with SA-PE and analyzed by flow cytometry.

Figure S4: Identity-divergence plots of the identified neutralizing antibodies. The 2D identity-divergence plots of the lineages of the neutralizing antibodies 1-0106, 1-0108, 1-11H2, 2-0126, 2-0139 and 2-01H5 in the context of the corresponding donor B cell repertoires. Sequences from NGS data are plotted as sequence identity (%, y axis) to the reference antibodies and sequence divergence (%, x axis) from their putative

germline genes. Color coding indicates sequence density on the 2D plot. The six neutralizing antibody lineages are visible as a distinct island of sequences with higher identity. The clones with germline divergence above 40% accounted for only less than 0.03% of total clones (367/3543964, 330/1916964 and 210/766607 for donor 1, donor 2 and the naïve donor, respectively).

Figures S5-8: Plot curves of antibody binning for RBD and S1 protein mutants. The title of each plot shows the antibody ID/mutant ID and KD (M) value of the corresponding antibody against the mutant.

Table S1

	Sex	Age
Donor 1	Male	43
Donor 2	Male	35

Table S2

	Donor 1			Donor 2	
	1st	2nd	3rd	1st	2nd
Input	1.35×10^{13}	1.60×10^{13}	1.90×10^{14}	1.75×10^{12}	2.48×10^{12}
Output	1.32×10^{6}	2.75×10^{6}	1.20×10^{9}	1.43×10^{6}	1.36×10^{8}
Output/Input	9.78×10^{-8}	1.72×10^{-6}	6.32×10^{-6}	8.17×10^{-7}	5.48×10^{-5}

Table S3

Antibody	SARS-CoV-2 strains (IC_{50} μg/mL)			
	QD01	P701	HN97	F13
1-0106	3.01	1.50	1.50	3.01
1-0108	16.29	8.14	10.86	16.29
1-11H2	20.83	15.63	15.63	31.25
2-0126	3.72	11.17	5.59	5.59
2-0139	5.74	5.74	3.83	11.48
2-01H5	2.40	0.80	1.20	2.40

Table S4

Mutant	Catalog number	Manufacturer
WT	SPD-C52H3	ACROBiosystems
R408I	SPD-S52H8	ACROBiosystems
W436R	SPD-S52H7	ACROBiosystems
D614G	40591-V08H3	Sino biological
V367F	SPD-S52H4	ACROBiosystems
N501Y	SPD-C52Hn	ACROBiosystems
Y453F	SPD-C52Hk	ACROBiosystems
N439K	SPD-C52Hg	ACROBiosystems
E484K	SRD-C52H3	ACROBiosystems
S477N	SPD-C52Hm	ACROBiosystems
HV69-70del	S1N-C52Hd	ACROBiosystems
HV69-70del, N501Y, D614G	S1N-C52Hk	ACROBiosystems
K417N, E484K,N501Y	SPD-C52Hp	ACROBiosystems

Figure S1

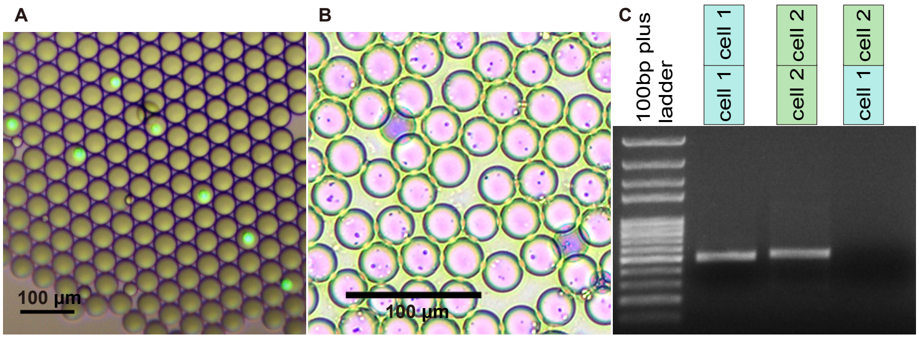

Figure S2

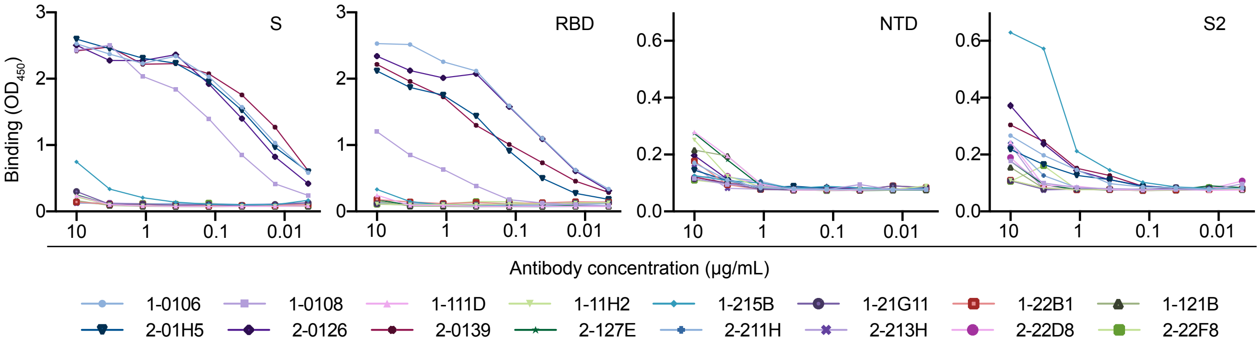

Figure S3

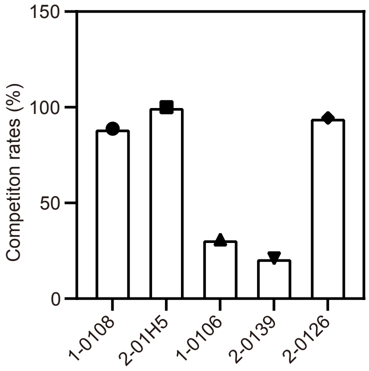

Figure S4

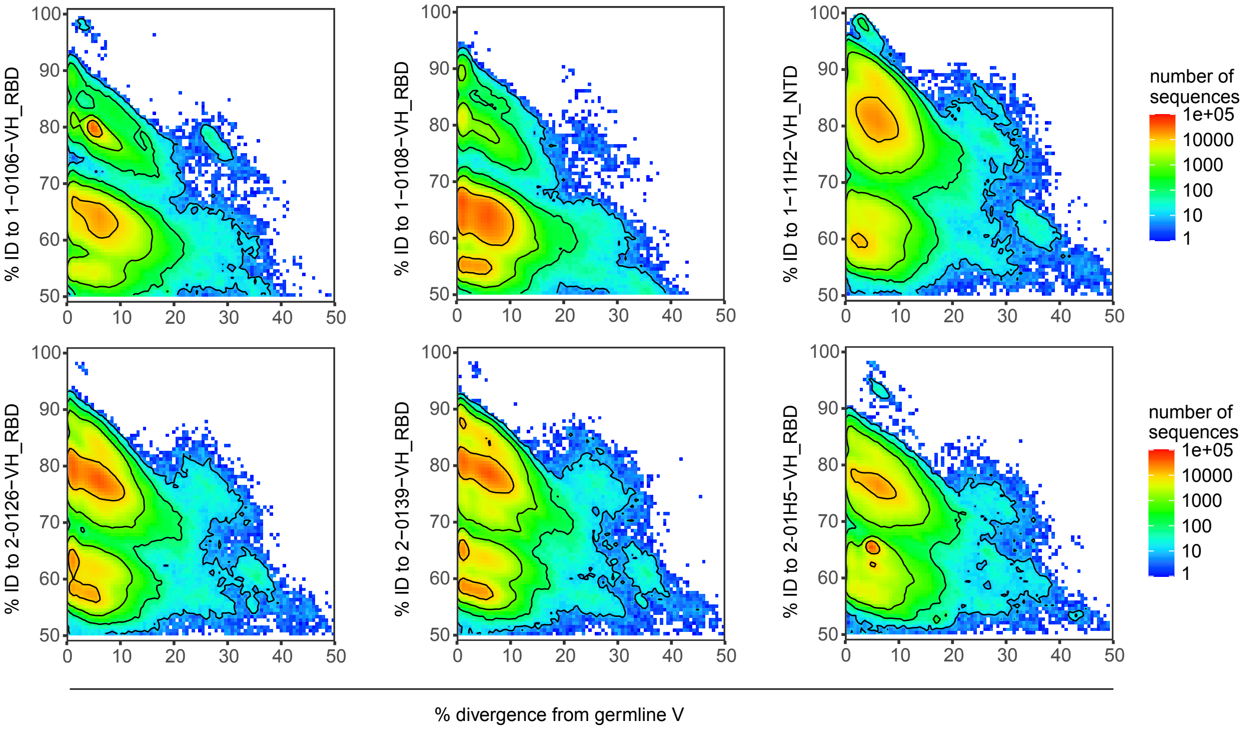

Figure S5

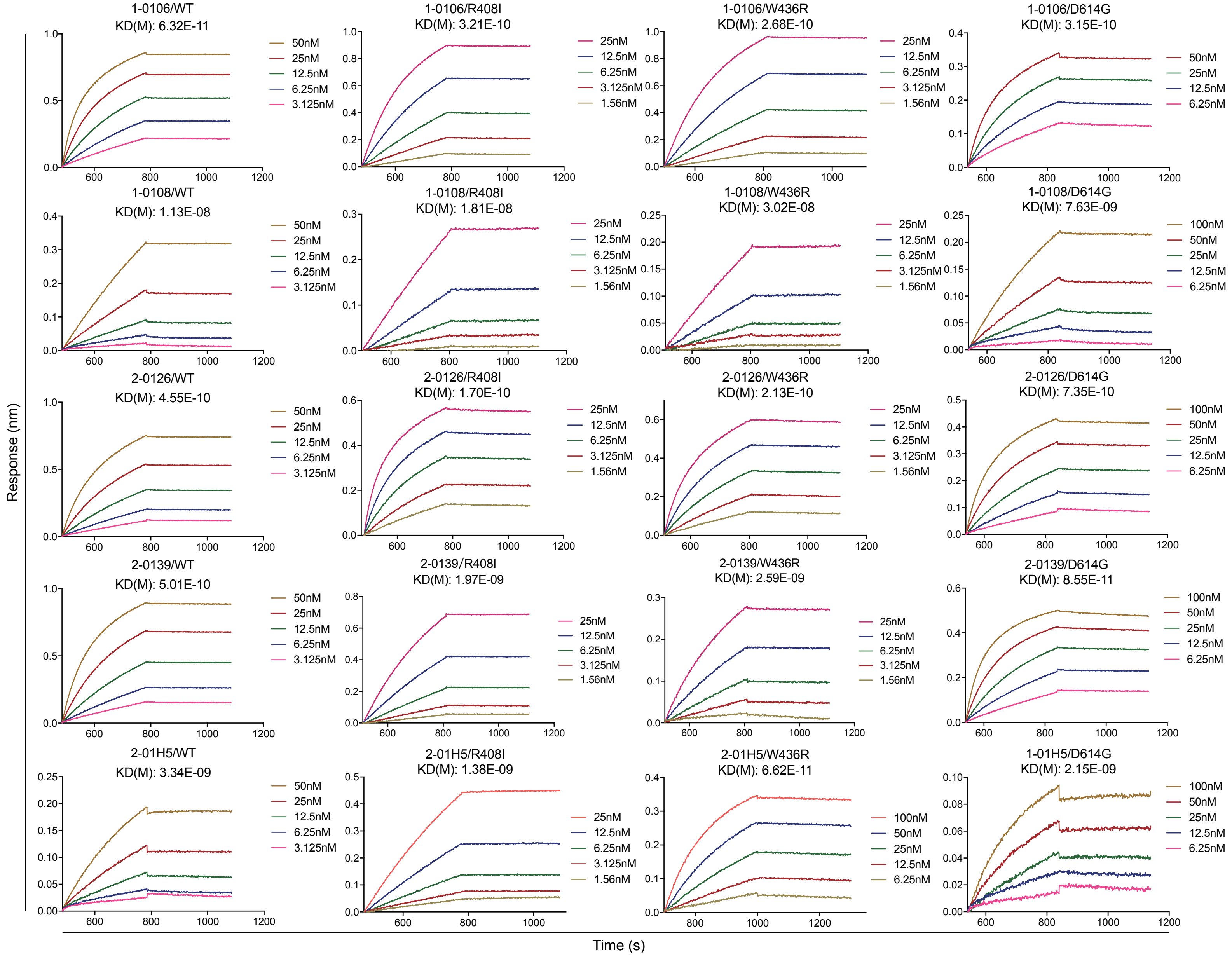
1-0106/WT
KD(M): 6.32E-11
1-0106/R408I
KD(M): 3.21E-10
1-0106/W436R
KD(M): 2.68E-10
1-0106/D614G
KD(M): 3.15E-10
1-0108/WT
KD(M): 1.13E-08
1-0108/R408I
KD(M): 1.81E-08
1-0108/W436R
KD(M): 3.02E-08
1-0108/D614G
KD(M): 7.63E-09
2-0126/WT
KD(M): 4.55E-10
2-0126/R408I
KD(M): 1.70E-10
2-0126/W436R
KD(M): 2.13E-10
2-0126/D614G
KD(M): 7.35E-10
2-0139/WT
KD(M): 5.01E-10
2-0139/R408I
KD(M): 1.97E-09
2-0139/W436R
KD(M): 2.59E-09
2-0139/D614G
KD(M): 8.55E-11
2-01H5/WT
KD(M): 3.34E-09
2-01H5/R408I
KD(M): 1.38E-09
2-01H5/W436R
KD(M): 6.62E-11
1-01H5/D614G
KD(M): 2.15E-09
Response (nm)
Time (s)

Figure S6

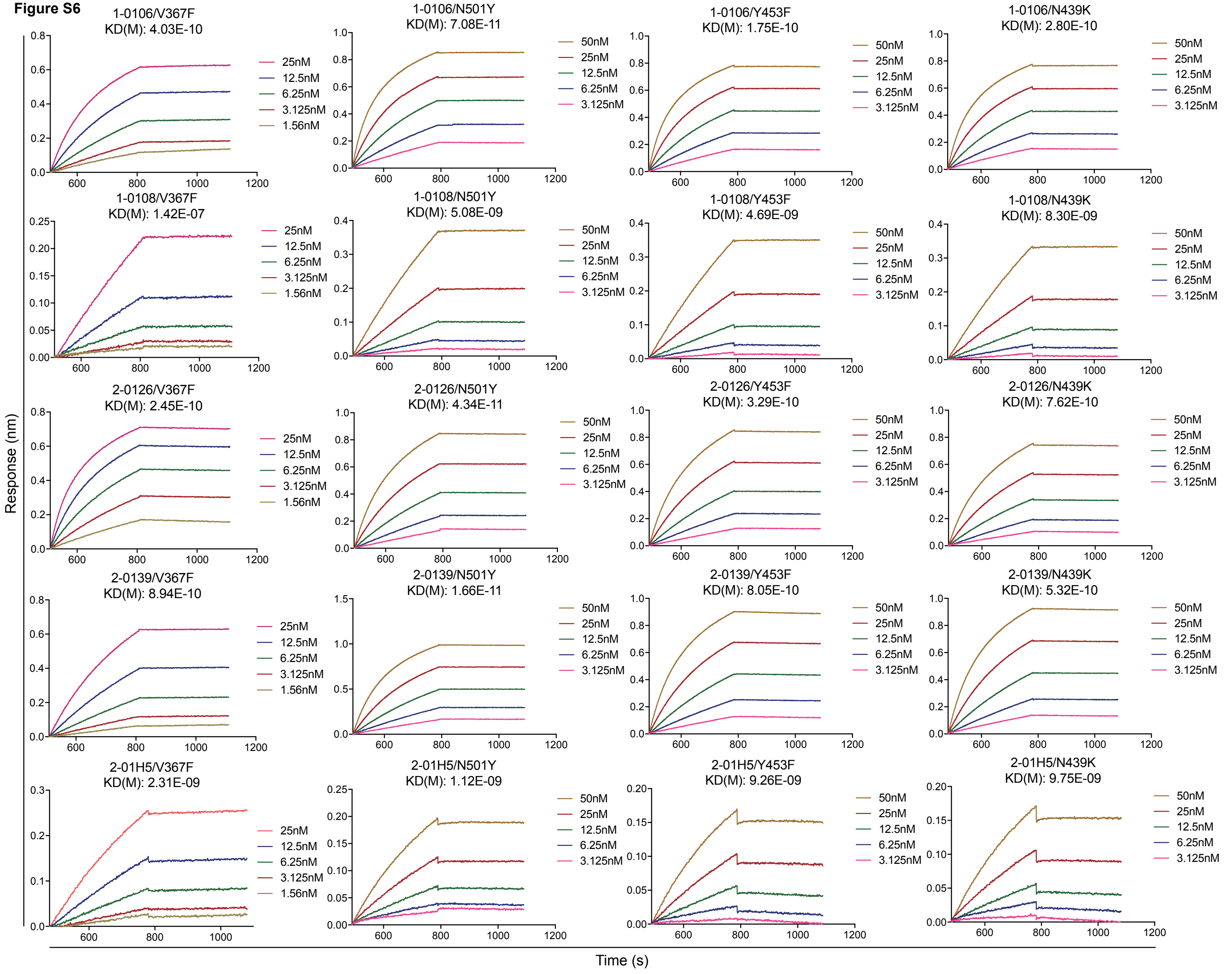

1-0106/V367F
KD(M): 4.03E-10
1-0106/N501Y
KD(M): 7.08E-11
1-0106/Y453F
KD(M): 1.75E-10
1-0106/N439K
KD(M): 2.80E-10
1-0108/V367F
KD(M): 1.42E-07
1-0108/N501Y
KD(M): 5.08E-09
1-0108/Y453F
KD(M): 4.69E-09
1-0108/N439K
KD(M): 8.30E-09
2-0126/V367F
KD(M): 2.45E-10
2-0126/N501Y
KD(M): 4.34E-11
2-0126/Y453F
KD(M): 3.29E-10
2-0126/N439K
KD(M): 7.62E-10
2-0139/V367F
KD(M): 8.94E-10
2-0139/N501Y
KD(M): 1.66E-11
2-0139/Y453F
KD(M): 8.05E-10
2-0139/N439K
KD(M): 5.32E-10
2-01H5/V367F
KD(M): 2.31E-09
2-01H5/N501Y
KD(M): 1.12E-09
2-01H5/Y453F
KD(M): 9.26E-09
2-01H5/N439K
KD(M): 9.75E-09
50nM
25nM
12.5nM
6.25nM
3.125nM
1.56nM
Response (nm)
Time (s)

Figure S7

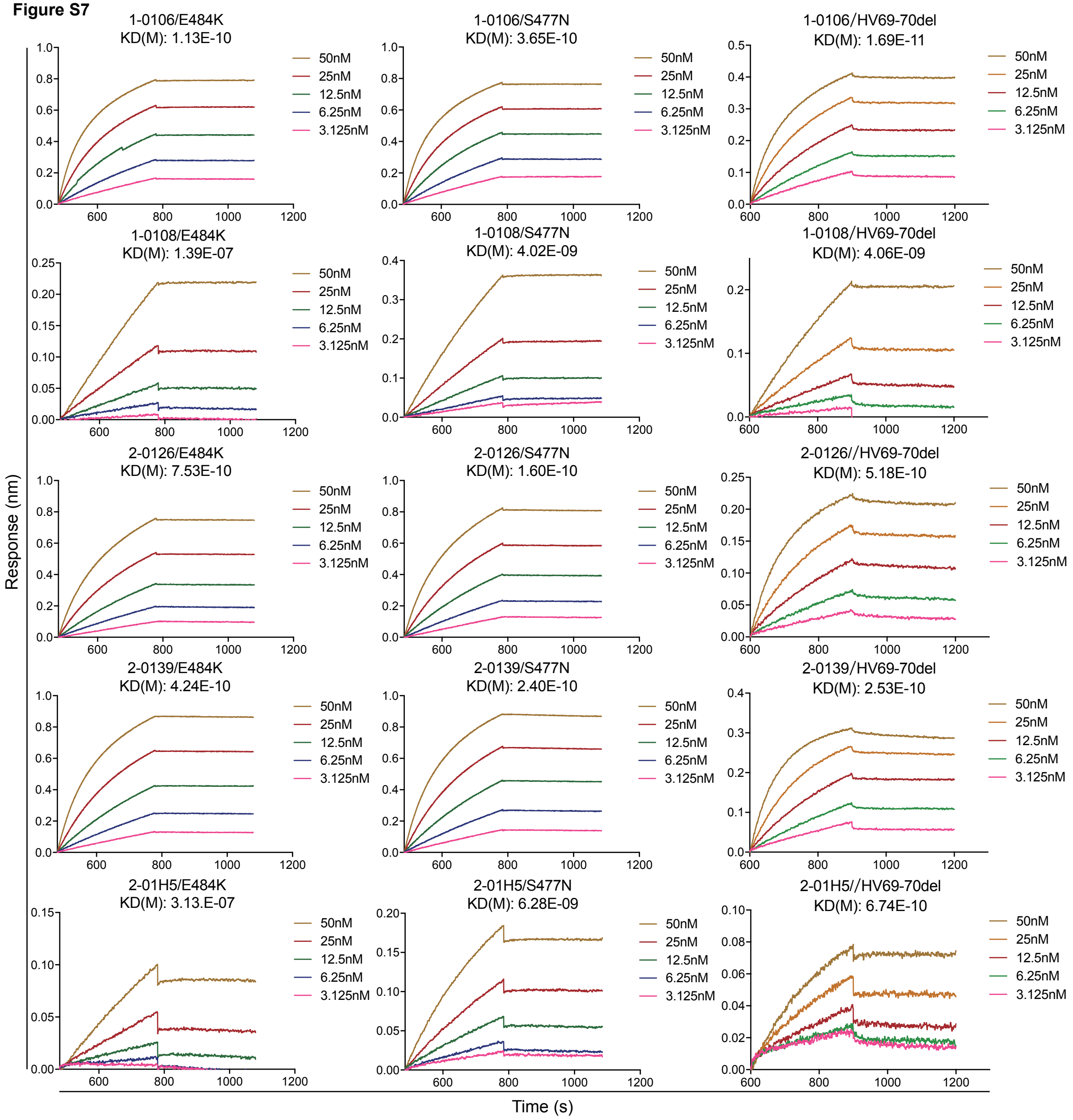
1-0106/E484K
KD(M): 1.13E-10
1-0106/S477N
KD(M): 3.65E-10
1-0106/HV69-70del
KD(M): 1.69E-11
1-0108/E484K
KD(M): 1.39E-07
1-0108/S477N
KD(M): 4.02E-09
1-0108/HV69-70del
KD(M): 4.06E-09
2-0126/E484K
KD(M): 7.53E-10
2-0126/S477N
KD(M): 1.60E-10
2-0126//HV69-70del
KD(M): 5.18E-10
2-0139/E484K
KD(M): 4.24E-10
2-0139/S477N
KD(M): 2.40E-10
2-0139/HV69-70del
KD(M): 2.53E-10
2-01H5/E484K
KD(M): 3.13.E-07
2-01H5/S477N
KD(M): 6.28E-09
2-01H5//HV69-70del
KD(M): 6.74E-10
50nM
25nM
12.5nM
6.25nM
3.125nM
Response (nm)
Time (s)

Figure S8

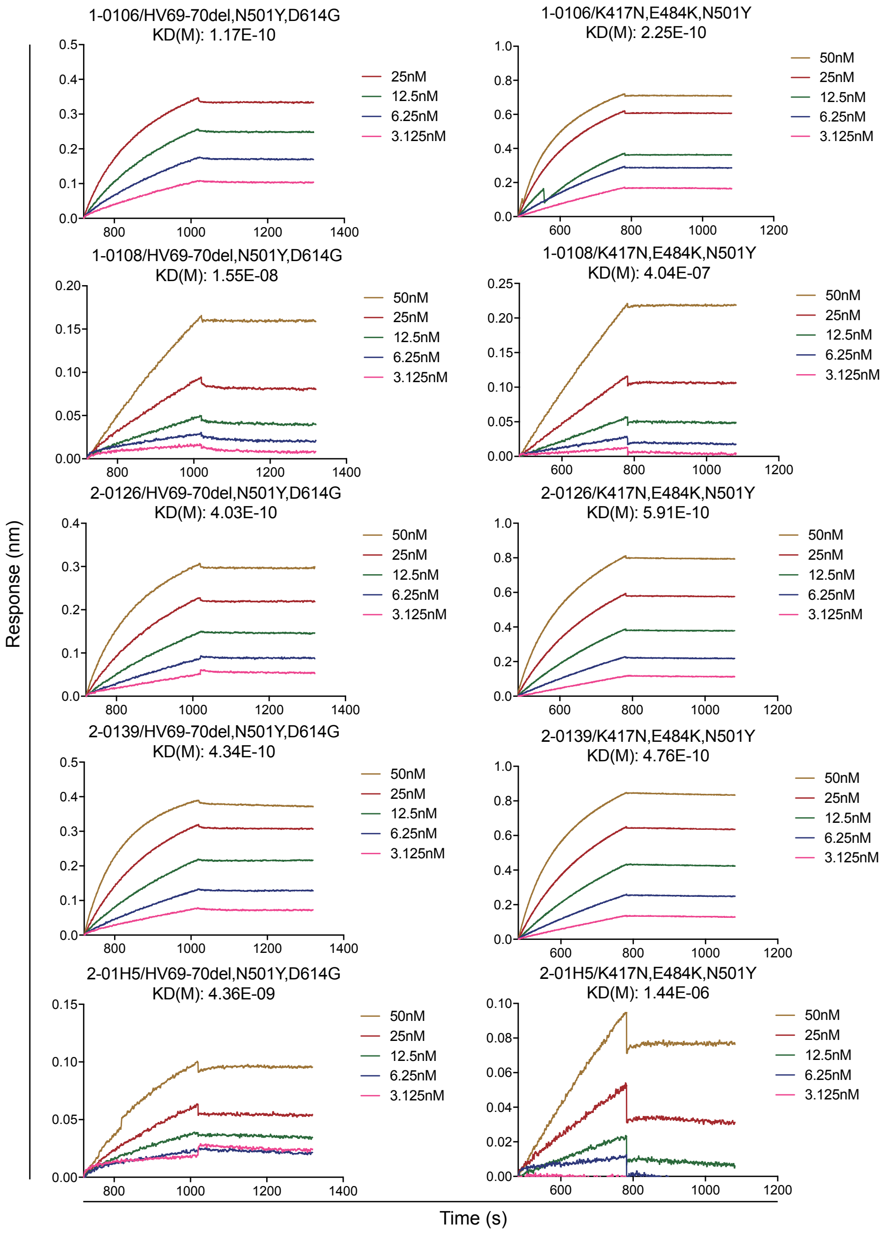
1-0106/HV69-70del,N501Y,D614G
KD(M): 1.17E-10
1-0106/K417N,E484K,N501Y
KD(M): 2.25E-10
1-0108/HV69-70del,N501Y,D614G
KD(M): 1.55E-08
1-0108/K417N,E484K,N501Y
KD(M): 4.04E-07
2-0126/HV69-70del,N501Y,D614G
KD(M): 4.03E-10
2-0126/K417N,E484K,N501Y
KD(M): 5.91E-10
2-0139/HV69-70del,N501Y,D614G
KD(M): 4.34E-10
2-0139/K417N,E484K,N501Y
KD(M): 4.76E-10
2-01H5/HV69-70del,N501Y,D614G
KD(M): 4.36E-09
2-01H5/K417N,E484K,N501Y
KD(M): 1.44E-06
50nM
25nM
12.5nM
6.25nM
3.125nM
Response (nm)
Time (s)
